# AngioVac System Used for Vegetation Debulking in a Patient with Tricuspid Valve Endocarditis: A Case Report and Review of the Literature

**DOI:** 10.1155/2017/1923505

**Published:** 2017-11-07

**Authors:** Hossam Abubakar, Ahmed Rashed, Ahmed Subahi, Ahmed S. Yassin, Mohamed Shokr, Mahir Elder

**Affiliations:** ^1^Department of Internal Medicine, Wayne State University, Detroit, MI, USA; ^2^Department of Cardiovascular Medicine, DMC/Wayne State University, Detroit, MI, USA

## Abstract

AngioVac is a vacuum-based device approved in 2014 for percutaneous removal of undesirable materials from the intravascular system. Although numerous reports exist with regard to the use of the AngioVac device in aspiration of iliocaval, pulmonary, upper extremity, and right-sided heart chamber thrombi, very few data are present demonstrating its use in treatment of right-sided endocarditis. In this case report, we describe the novel device used in debulking a large right-sided tricuspid valve vegetation reducing the occurrence of septic embolisation and enhancing the efficacy of antibiotics in clearance of bloodstream infection. Further research is needed in larger RSIE patient populations to confirm the benefits and the potential of improved outcomes associated with the AngioVac device as well as identify its potential complications.

## 1. Case Presentation and Procedure Description

**Figure 1:**
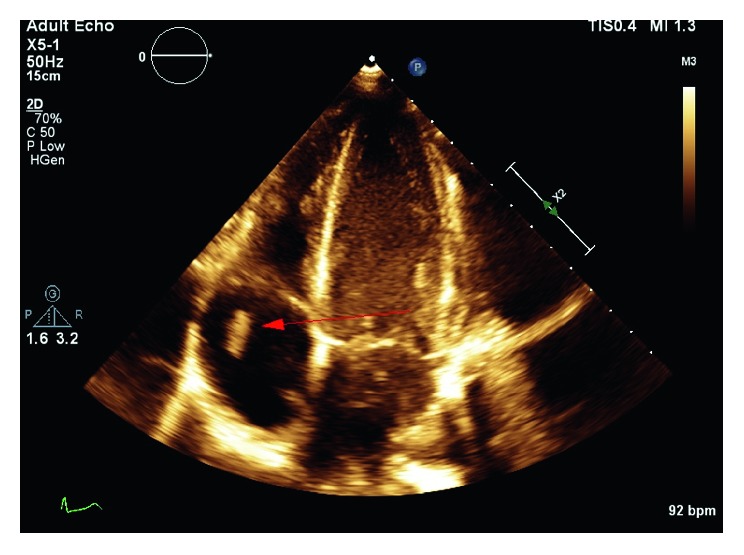
Preprocedural transthoracic echocardiogram (TTE). Apical 4-chamber view revealing a 3 × 1.5 cm tricuspid valve vegetation (red arrow).

A 33-year-old female with a history of hepatitis C and active intravenous heroin use presented to an outside facility with complaints of worsening shortness of breath. A transthoracic echocardiogram (TTE) was done and showed tricuspid valve (TV) vegetation measuring 3 × 1.5 cm with severe tricuspid regurgitation (TR). CT scan of the chest revealed signs concerning septic pulmonary emboli. She was also found to have severe sepsis and was transferred to the coronary care unit for further management. Blood cultures on admission yielded *Streptococcus pyogenes*, and she was empirically started on vancomycin, linezolid, and cefepime until organisms were shown to be pan-sensitive after which antibiotic coverage was narrowed to penicillin G. Clindamycin was added for synergism, given her extensive septic pulmonary emboli. Repeat blood cultures revealed the same organism for 3 consecutive days. Her respiratory status continued to worsen, and she was consequently intubated. No source other than intravenous drug use (IVDU) was found to be the cause of the bacteremia. Due to the size of the vegetation and persistent bacteremia despite appropriate medical therapy, cardiothoracic surgery was consulted for the possibility of surgical intervention, but the patient was deemed a poor surgical candidate due to her poor nutritional state and hemodynamic instability. After discussion with the family regarding the patient's critically ill state and poor prognosis, a decision was made to transfer her to our facility for debulking of the tricuspid valve vegetation using the AngioVac system. A repeat TTE at our facility confirmed prior findings of TV vegetation measuring 3 × 1.5 cm and moderate TR (Figure 1). Blood cultures repeated at our facility revealed no growth. The patient was immediately taken to the catheterization laboratory for vegetation debulking using the AngioVac system.

**Figure 2:**
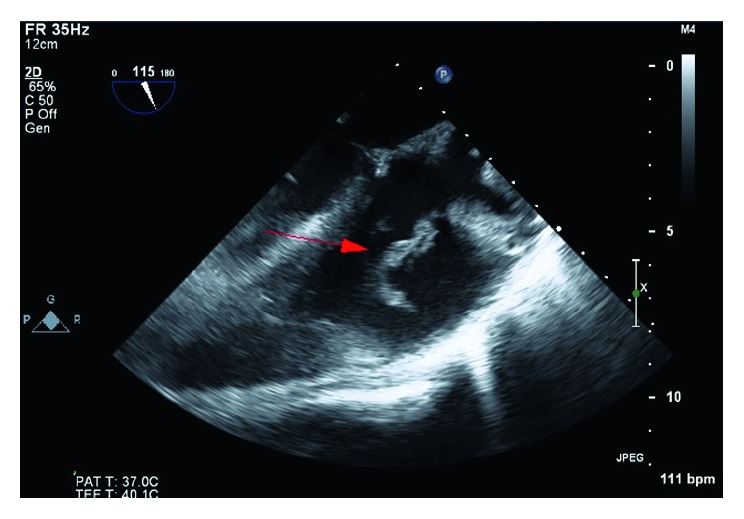
Preprocedural transesophageal echocardiogram (TEE). Midesophageal longitudinal-axis 115-degree view revealing the tricuspid valve vegetation 4.2 cm in its largest diameter (red arrow).

**Figure 3:**
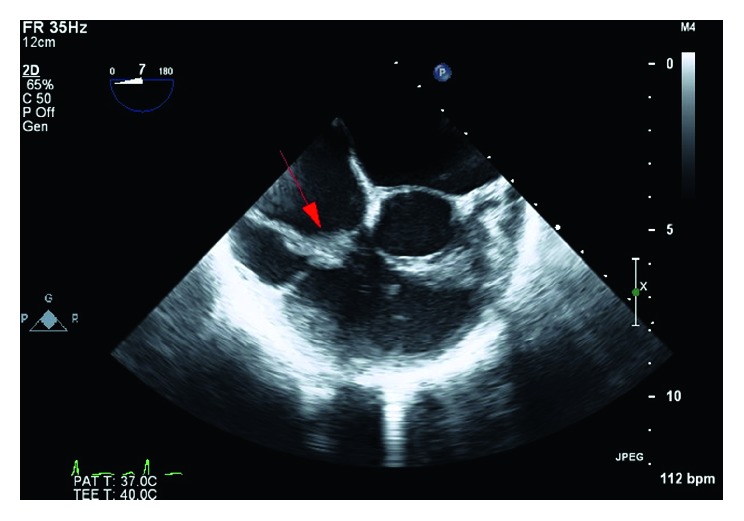
Postprocedural transesophageal echocardiogram (TEE). Midesophageal short-axis 0-degree view revealing the tricuspid valve vegetation reduced in size to 2.1 cm from its largest diameter (red arrow).

The patient arrived in the catheterization laboratory intubated and sedated. Bilateral femoral veins were accessed using the modified Seldinger technique. A 6-French (Fr) precision sheath was placed in the right common femoral vein, and an 8-French sheath was inserted into the left common femoral vein. A 6-French sheath was inserted into the right internal jugular vein (IJ). The right IJ was then progressively dilated with escalating size sheaths and, subsequently, a 24-French sheath was placed over a Lunderquist wire. The left common femoral vein sheath was escalated to a 16-French outlet sheath. The AngioVac system was advanced through the right IJ—a 24-French inlet sheath over a Wholey wire. Under transesophageal echocardiography (TEE) guidance, the system was further advanced via the superior vena cava and right atrium towards the TV, where the vegetation was then repeatedly suctioned. Two pieces of the vegetation were successfully extracted. The intraprocedural TEE revealed a 50–60% reduction in the size of the TV vegetation (2.1 cm decrease in the longest dimension) (Figures 2 and 3). The AngioVac system was removed, and hemostasis was achieved in both the right IJ and the left common femoral venous accesses via purse-string sutures. The patient tolerated the procedure well. Heparin was used for anticoagulation during the procedure.

The patient's condition improved, and she was extubated on the second day after the procedure. Antibiotics therapy continued. Subsequent blood cultures remained negative, and she became afebrile with a steady decline in her white blood cell count. Seven days after the AngioVac procedure, she was discharged to a rehabilitation facility to finish six weeks of intravenous penicillin G therapy. During a follow-up appointment at an infectious disease clinic one month after hospital discharge, the patient was seen with no symptoms or signs of reinfection.

## 2. Discussion

The vast majority of cases of right-sided infective endocarditis (RSIE) are in the intravenous drug use (IVDU) population [1]. Epidemiological studies have shown that, among IVDU patients who present with fever, 13% will have echocardiographic evidence of IE [2] and 41% of bacteremic IVDU patients will have evidence of IE, the majority of which is RSIE [3]. Clinical features are distinct from those of left-sided endocarditis. The most common manifestations are fever and sepsis in addition to symptoms related to septic pulmonary emboli including chest pain, dyspnea, cough, and hemoptysis [4]. Although the Duke criteria have their theoretical limitations when applied in diagnosing RSIE, they remain the most widely utilized criteria for diagnosis [4]. The close association of the disease to IVDU creates obstacles to successful treatment. These challenges include nonadherence to in-hospital treatment and noncompliance to follow-up, creating scarcity of data on long-term outcomes [1]. Despite these challenges, RSIE is shown to have a relatively more favorable prognosis when compared to left-sided disease with an in-hospital mortality of less than 10% [1].

Management is multidisciplinary and involves the involvement of a general cardiologist, an infectious disease specialist, a cardiac surgeon, and, occasionally, an interventional cardiologist. There is sufficient evidence that, in uncomplicated cases of RSIE caused by *Staphylococcus aureus*, two weeks of antibiotic therapy is sufficient [5]. Antibiotic treatment of nonstaphylococcal RSIE, as in our case, is analogous to that of left-sided disease and includes an extended treatment course of 4–6 weeks [6]. While indications of surgery for left-sided disease are well established, the role of surgery in RSIE is less clear. Nevertheless, there are complications where surgery is agreed to be reasonable. These include right heart failure secondary to severe tricuspid regurgitation with poor response to medical therapy, sustained infection caused by difficult-to-treat organisms (i.e., fungi and multidrug-resistant bacteria) or lack of response to appropriate antimicrobial therapy, and tricuspid valve vegetations that are ≥ 20 mm in diameter and recurrent pulmonary embolism despite antimicrobial therapy [6]. When surgery is warranted, valve repair rather than replacement is preferred especially in IVDU patients due to the risk of recurrence of infection in prosthetic valves [6]. Our patient had a tricuspid valve vegetation with a diameter of >30 mm, which led to the consideration of surgical intervention. Due to the patient's poor nutritional status, respiratory failure due to multiple septic emboli, and early signs of DIC, she was deemed an unsuitable candidate for open cardiac surgery.

A noninvasive approach utilizing the AngioVac device was justified, given our patient's critically ill state and high perioperative risk. The US Food and Drug Administration approved the AngioVac system for removal of unwanted intravascular materials (thrombi and emboli) in 2014 [7]. The device is composed of a venous drainage cannula and a reinfusion (venous return) cannula which are connected to an extracorporeal circuit and a commercially available pump head and bubble trap. The venous drainage component is a 22 Fr cannula with a funnel-shaped distal tip that can be advanced through a 26 Fr sheath over a guidewire into the venous system percutaneously. When the bypass pump is started, a suction force is created that facilitates aspiration of blood and thrombotic materials into the tip of the AngioVac cannula, circulating the blood through a filter. After filtration, the drained blood is returned to the patient via a second percutaneously placed reinfusion venous cannula through the internal jugular or femoral vein [8]. This recirculation of venous blood minimizes intraprocedural blood loss and the need of blood transfusion. There are numerous reports on the use of the AngioVac device in aspiration of iliocaval, pulmonary, upper extremity, and right-sided heart chamber thrombi [8]. In contrast to alternative percutaneous thrombectomy devices that require prior thrombus fragmentation and/or thrombolysis to facilitate aspiration, the AngioVac device has the advantage of aspirating the whole intact thrombus. This eliminates the need of preaspiration thrombolysis and reduces the consequent risk of embolisation [9].

Very few data are present with regard to the use of the AngioVac device management of RSIE. The biggest study to date by George et al. reviewed the periprocedural course of 33 patients with tricuspid valve (TV) endocarditis who underwent TV vegetation debulking using the AngioVac device. Average preprocedural vegetation size was 2.1 ± 0.7 cm and average vegetation size on postprocedural echocardiography was 0.82 ± 0.5 cm, demonstrating a 61% average reduction in vegetation size that is in parallel to that seen in our patient. All patients in the study survived the procedure, and 90.9% survived the hospitalization with no further reinfection. We searched two literature databases (PubMed and Embase) to identify publications reporting the use of the AngioVac device for treatment of RSIE (results in Table 1). Most reported indications for the use of the AngioVac device for vegetation debulking in RSIE are consistent with AHA proposed indications of surgery [6]. However, there are reports on utilization of the AngioVac system to debulk lead vegetations as a bridge to percutaneous lead removal in patients with cardiovascular implantable electronic device- (CIED-) related endocarditis [10–12]. Despite data that demonstrate the safety of percutaneous lead removal in patients with vegetations >1 cm [13], the risk of septic pulmonary embolism remains significant at 34–55% in this subset of patients [14, 15]. This predisposes to further infectious complications including pulmonary abscesses and refractory sepsis. Although large population data are lacking, Patel et al. proposed that the AngioVac device used for large vegetation debulking prior to percutaneous lead removal may reduce the incidence of septic pulmonary embolism in patients with CIED-related endocarditis [11]. The use of the novel technique has also been reported as a bridge to reduce lag time and perioperative complications of cardiac surgery [7, 16]. Diminishing intracardiac vegetation size and the associated bacterial load may increase the efficacy of antibiotics in clearing of the bloodstream infection. This concept is supported by studies investigating the efficacy of antibiotics on different bacterial inoculums [17]. High bacterial inoculums are associated with higher antibiotic resistance and reduced penetration, a phenomenon known as the inoculum effect [18]. Through vegetation debulking, the AngioVac allows for reduction of the bacterial inoculum and enhancement of antibiotic activity. This hastens resolution of the septic state and consequent hemodynamic compromise which is a key determinant of operative and postoperative mortality associated with cardiac surgery [19].

## 3. Conclusion

Our case describes a novel modality of treatment in RSIE. Although currently approved for removal of intravascular thrombi and emboli, the AngioVac device may be a promising noninvasive treatment option for RSIE for a multitude of indications. The novel modality may be used as a substitute for surgery where surgery is indicated but not possible due to associated perioperative risk. The device may also be utilized as a bridge to surgery and percutaneous CIED removal by reducing the perioperative risk through hastening clearance of infection, improving hemodynamics, and reducing the risk of periprocedural septic pulmonary embolism. Further research is needed in larger RSIE patient populations to confirm the benefits and the potential of improved outcomes associated with the AngioVac device as well as identify its potential complications.

## Figures and Tables

**Table 1:** Reports on the use of the AngioVac device in treatment of RSIE.

Author/year	Type of publication	Age/sex	Organism	Location of vegetation	Indication for procedure	Preprocedural vegetation size	Reduction in vegetation size	Postprocedural bacteremia	Tricuspid regurgitation (TR) progression	Outcome
Todoran et al. [20] (2011)	Case report	53 y/o, M	*Haemophilus parainfluenzae*	SVC/RA junction	Lack of response to appropriate antimicrobial therapy	1.7 cm	100% removal	Resolved	Not reported	Improvement with no further sequel

Jones et al. [21] (2017)	Case report	25 y/o, F	*Candida albicans*	(i) SV/RA junction attached to ICD RV lead	(i) Persistent fungemia despite appropriate antimicrobial therapy	SV/RA junction: 6.1 cm × 1.65 cm	Removal of 6 cm vegetation (residual vegetation size not reported)	Resolved	Not reported	Improvement with no further sequel
				(ii) RA	(ii) Recurrent septic emboli	RA: −2.1 cm × 1.6 cm				

Schaerf et al. [10] (2016)	Retrospective study (20 patients)	Mean age: 76 ± 11	8 coagulase-negative SA	13 ICD; 7 pacemaker	(i) Lack of response to appropriate antimicrobial therapy	Average size: 3.6 cm ± 1.2 cm	Not mentioned	Resolved in 19/20 patients	Not reported	Not reported clearly
			3 MSSA							
			4 MRSA							
			3 Streptococci							
		Sex not reported								
			1 *Enterococcus*							
			1 Polymicrobial							
					(ii) Bridge to percutaneous lead removal					

Thiagaraj et al. [22] (2017)	Case series	Patient 1: 35 y/o, M	MSSA	SVC/RA junction extending into TV	(i) Lack of response to appropriate antimicrobial therapy	4.5 cm	100% removal	Resolved	Not reported	Improvement with no further sequel
					(ii) Vegetation size ≥ 20 mm					
		Patient 2: 28 y/o, F	MRSA	TV	(i) Lack of response to appropriate antimicrobial therapy	2.2 × 1.7 cm	100% removal	Resolved	Not reported	MRSA bacteremia recurrence, cardiac arrest, and death 5 days post procedure
					(ii) Vegetation size ≥ 20 mm					
		Patient 3: 53 y/o, F	*Enterococcus faecalis*	Bioprosthetic TV	(i) Vegetation size ≥ 20 mm	3.2 cm	25–50% reduction in size	Resolved	Improvement from moderate to mild	Improvement with mild worsening of TR
					(ii) Worsening of TV regurgitation					

Divekar et al. [7] (2013)	Case report	17 y/o, M	MSSA	Pulmonary valve	Recurrent pulmonary embolism despite antimicrobial therapy	3.5 cm × 1.5 cm	Significant reduction (residual vegetation size not reported)	Resolved	Not reported	Clinical improvement with no further sequel

George et al. [23] (2017)	Retrospective study (33 patients)	Mean age: 37 ± 12	14 MRSA	TV	Lack of response to appropriate antimicrobial therapy	2.1 cm ± 0.7 cm	Average of 61% reduction in size	Resolved in 28/33 patients	14 patients: worsening of TR (3 required elective TV repair)	28 patients: improvement with no further sequel
			11 MSSA							
		12, M	3 polymicrobial							1 patient: developed postprocedural cardiac tamponade requiring pericardiocentesis
		21, F	5 *Candida*							3 patients: death

Makdisi et al. [16] (2016)	Case report	24 y/o, M	MRSA	TV	Lack of response to appropriate antimicrobial therapy	0.9 cm × 0.7 cm	80% reduction in size	Resolved	No change	Clinical improvement with no further sequel
						0.7 cm × 1 cm				

Patel et al. [11] (2013)	Case series	Patient 1: 59 y/o, M	SA	ICD lead	(i) Vegetation size ≥ 20 mm	3 cm × 2 cm	Significant reduction (residual vegetation size not reported)	Resolved	Improvement in degree of TR	Clinical improvement with no further sequel
					(ii) Bridge to percutaneous lead removal					
		Patient 2: 82 y/o, M	Group B *Streptococcus*	(i) Pacemaker lead	(i) Vegetation size ≥ 20 mm	(i) Pacemaker lead: 4 cm × 1.5 cm	Significant reduction (residual vegetation size not reported)	Not reported	Not reported	Not reported
				(ii) TV	(ii) Bridge to percutaneous lead removal	(ii) TV: 0.5 cm × 1.1 cm				
		Patient 3: 56 y/o, F	MRSA	Pacemaker lead	(i) Vegetation size ≥ 20 mm	3.5 cm × 1.7 cm	Significant reduction (residual vegetation size not reported)	Persistent bacteremia	Worsening of TR	Formation of new vegetation with severe TR that required TV repair
					(ii) Bridge to percutaneous lead removal					

Dalia et al. [12] (2016)	Case report	26 y/o, F	Not reported	TV	Bridge to pulmonary artery aneurysm repair	1.6 cm × 0.8 cm	Significant reduction (residual vegetation size not reported)	Not reported	Not reported	Underwent pulmonary artery aneurysm repair successfully; clinical improvement with no further sequel

Hosoba et al. [24] (2015)	Case series	Patient 1: 67 y/o, F	MRSA	RA	Not reported	1.5 cm × 1.5 cm	Not reported	Resolved	Not reported	Clinical improvement with no further sequel
		Patient 2: 33 y/o, M	*Enterobacter cloacae*	RA near Chiari network	Not reported	2.2 cm × 0.6 cm	Not reported	Resolved	Not reported	Clinical improvement with no further sequel
		Patient 3: 70 y/o, M	MSSA	SVC/RA junction	Vegetation size 20 mm	3.4 cm × 1.3 cm	Not reported	Resolved	Not reported	Clinical improvement with no further sequel

M: male, F: female, y/o: years old, SVC: superior vena cava, RA: right atrium, ICD: implantable cardioverter defibrillator, RV: right ventricle, SA: *Staphylococcus aureus*, MSSA: methicillin-sensitive *Staphylococcus aureus*, MRSA: methicillin-resistant *Staphylococcus aureus*, TV: tricuspid valve, TR: tricuspid regurgitation.
